# A randomized phase II study of capecitabine-based chemoradiation with or without bevacizumab in resectable locally advanced rectal cancer: clinical and biological features

**DOI:** 10.1186/s12885-015-1053-z

**Published:** 2015-02-26

**Authors:** Ramon Salazar, Jaume Capdevila, Berta Laquente, Jose Luis Manzano, Carles Pericay, Mercedes Martínez Villacampa, Carlos López, Ferran Losa, Maria Jose Safont, Auxiliadora Gómez, Vicente Alonso, Pilar Escudero, Javier Gallego, Javier Sastre, Cristina Grávalos, Sebastiano Biondo, Amalia Palacios, Enrique Aranda

**Affiliations:** 1 Catalan Institute of Oncology (ICO), Bellvitge Biomedical Research Institute (IDIBELL), L’Hospitalet de Llobregat, Barcelona, Spain; 2 Medical Oncology, Hospital Universitari Vall D’Hebrón, Barcelona, Spain; 3 Medical Oncology, Hospital Universitari German Trias I Pujol, Barcelona, Spain; 4 Medical Oncology, Complejo Sanitario Parc Taulí, Barcelona, Spain; 5 Medical Oncology, Hospital Universitario Marqués de Valdecilla, Santander, Spain; 6 Medical Oncology, Hospital General de L’Hospitalet, Barcelona, Spain; 7 Medical Oncology, Hospital General Universitario de Valencia, Valencia, Spain; 8 Medical Oncology, Reina Sofía Hospital, University of Córdoba, Maimonides Institute of Biomedical Research (IMIBIC); Spanish Cancer Network (RTICC), Instituto de Salud Carlos III, Córdoba, Spain; 9 Medical Oncology, Hospital Universitario Miguel Servet, Zaragoza, Spain; 10 Medical Oncology, Hospital Clínico Universitario Lozano Blesa, Zaragoza, Spain; 11 Medical Oncology, Hospital General U. de Elche, Alicante, Spain; 12 Medical Oncology, Hospital Clínico Universitario San Carlos, Madrid, Spain; 13 Medical Oncology, Hospital Doce de Octubre, Madrid, Spain; 14 General and Digestive Surgery Hospital Universitario de Bellvitge, Barcelona, Spain; 15 Radiation Oncology, Hospital Universitario Reina Sofía, Córdoba, Spain

**Keywords:** Bevacizumab, Chemoradiotherapy, Locally-advanced, Rectal cancer, Resectable

## Abstract

**Background:**

Perioperatory chemoradiotherapy (CRT) improves local control and survival in patients with locally advanced rectal cancer (LARC). The objective of the current study was to evaluate the addition of bevacizumab (BEV) to preoperative capecitabine (CAP)-based CRT in LARC, and to explore biomarkers for downstaging.

**Methods:**

Patients (pts) were randomized to receive 5 weeks of radiotherapy 45 Gy/25 fractions with concurrent CAP 825 mg/m^2^ twice daily 5 days per week and BEV 5 mg/kg once every 2 weeks (3 doses) (arm A), or the same schedule without BEV (arm B). The primary end point was pathologic complete response (ypCR: ypT_0_N_0_).

**Results:**

Ninety pts were included in arm A (44) or arm B (46). Grade 3–4 treatment-related toxicity rates were 16% and 13%, respectively. All patients but one (arm A) proceeded to surgery. The ypCR rate was 16% in arm A and 11% in arm B (*p* =0.54). Fifty-nine percent *vs* 39% of pts achieved T-downstaging (arm A *vs* arm B; *p* =0.04). Serial samples for biomarker analyses were obtained for 50 out of 90 randomized pts (arm A/B: 22/28). Plasma angiopoietin-2 (Ang-2) levels decreased in arm A and increased in arm B (*p* <0.05 at all time points). Decrease in Ang-2 levels from baseline to day 57 was significantly associated with tumor downstaging (*p* =0.02).

**Conclusions:**

The addition of BEV to CAP-based preoperative CRT has shown to be feasible in LARC. The association between decreasing Ang-2 levels and tumor downstaging should be further validated in customized studies.

**Trial registry:**

Clinicaltrials.gov identifier NCT01043484. Trial registration date: 12/30/2009.

## Background

Surgery is the mainstay of curative therapy for patients with rectal cancer confined to the bowel and regional lymph nodes. Nevertheless, rectal cancers have a high incidence of local failure. Recurrent pelvic disease is associated with significant morbidity and substantially shorter survival [1]. Perioperatory chemoradiotherapy (CRT) improves local control and survival in patients with locally advanced (T3-T4) rectal cancer (LARC) [2]. Moreover, this strategy maximizes downstaging, increases the rate of sphincter-sparing surgery and provides early exposure to systemic therapy.

Capecitabine is being integrated into the treatment of patients with colorectal cancer as an alternative to 5-fluorouracil (5-FU), resulting in improved convenience without compromising efficacy [3]. In addition, radiation induces thymidine phosphorylase and enhances the efficacy of capecitabine, leading to a synergistic effect [4]. A recent non-inferiority phase III study has shown capecitabine can replace 5-FU in adjuvant or neoadjuvant CRT regimens for patients with LARC [5].

In rectal cancer, several trials of bevacizumab with chemoradiation have shown promising results [6-11], but the lack of randomization and the bias associated with single-arm trials raises important concerns when interpreting these data. No randomized study to date has tested the use of bevacizumab in the neoadjuvant setting for localized disease.

Based on our previous experience (back to back submission [12]) and reports of preliminary feasibility of bevacizumab and chemoradiation in rectal cancer, we conducted a randomized trial of neoadjuvant bevacizumab and chemoradiation in patients with resectable LARC. Also, as little data exists on the role of biomarkers as predictors of response to bevacizumab when adding to preoperative CRT in LARC, we explored potential biomarkers that have been previously found to change in response to bevacizumab in other translational trials [11,13-20].

The following possible prognostic factors for tumor angiogenesis were evaluated: vascular endothelial growth factor (VEGF) and circulating soluble VEGF receptor 2 (VEGFR-2) which can be expressed in malignant tumors [21], angiopoietin-2 (Ang-2) expression, a molecule which promotes destabilization of blood vessels, and whose expression decreases along with microvessel density (MVD) after bevacizumab administration supporting the theory on the normalization of the vessels postulated for that drug [18], and the intratumor MVD since an increase in the number of tumor vessels might constitute a higher risk to develop metastasis [22-25].

## Methods

This open, multicenter randomized phase II trial was carried out by the Spanish Cooperative Group for the Treatment of Digestive Tumors (TTD group). The study was conducted in accordance with the Declaration of Helsinki and Good Clinical Practice Guidelines. Before starting the study, written informed consent was obtained from all patients in the study. The protocol was approved by the institutional review boards of all participating centers. Reference Ethic Committee: Comité Ético de Investigación Clínica del Hospital Universitario 12 de Octubre, Avda de Córdoba, s/n, 28041 Madrid.

### Patient selection

Patients 18 years of age or older with locally advanced rectal adenocarcinoma, clinical stage II-III [American Joint Committee on Cancer version 6: pelvic magnetic resonance imaging (MRI) was used to define T category and N category], within <15 cm from the anal verge, and an Eastern Cooperative Oncology Group (ECOG) performance status of 0 or 1 were eligible. All patients were required to be candidates for definitive surgical resection. Patients had adequate bone marrow and organ function and no previous chemotherapy or radiation for rectal cancer. Exclusion criteria included uncontrolled hypertension, clinically significant cardiac disease, having undergone major surgery within 28 d of trial therapy, recent or current use of full-dose oral or parenteral anticoagulants or thrombolytic agents, chronic daily treatment with high-dose aspirin, or treatment with nonsteroidal anti-inflammatory drugs.

### Treatment schedule

Patients were randomly allocated in a 1:1 ratio to CRT treatment with or without bevacizumab, using permuted blocks with stratification by center and tumor location (upper or middle third *vs* lower third). Radiotherapy (RT) consisted of a total of 45 Gy delivered in 25 daily fractions over 5 weeks (1.8 Gy/d for 5 d/wk). Patients in arm A received concomitant bevacizumab (5 mg/kg) on day 1 of weeks 1, 3, and 5, plus capecitabine (825 mg/m^2^) twice daily concomitant with RT; the same schedule without bevacizumab was administered to patients in arm B. One cycle was considered two weeks for cycle 1 and 2, and one week for cycle 3. Standard surgery, , was performed 6–8 weeks after the completion of CRT. A radical resection of the rectal tumor along with an appropriate vascular pedicle and accompanying lymphatic drainage was made. For tumors in the mid and lower rectum total mesorectal excision (TME) was carried out. However, for tumors in the upper rectum (at or above 10 cm from the anal margin) the mesorectum was resected at 5 cm or more distal to the tumor. Postoperative adjuvant chemotherapy was administered at the investigators discretion.

The protocol stipulated detailed capecitabine dose-modification criteria according to toxicity, graded using the National Cancer Institute Common Toxicity Criteria (NCI-CTC) version 3.0. No dose reductions for bevacizumab were planned.

### Evaluations during the study

Pretreatment evaluation included a complete medical history and physical examination, hematology with differential leucocyte count, chemistry, coagulation profile, urinalysis, carcinoembryonic antigen, electrocardiogram, complete colonoscopy with biopsy, abdominal and thoracic computerized axial tomography (or thoracic x-ray), and pelvic MRI. Medical history, physical examination, and laboratory studies were repeated prior to the start of each treatment cycle (days 1, 15 and 29 ± 2 days). After surgery, histologic tumor infiltration (ypTypN) and grading of regression [assessed using the Mandard scale [26]] were evaluated.

All patients were scheduled for a follow-up period of 5 years after surgery.

### Biologics evaluation

Participation in the biologic sub-study was optional. To those patients who gave their consent to have the biomarkers analyzed, plasma levels of VEGF, VEGFR-2, and Ang-2 were measured at baseline (d1, pre-treatment), 15 (d15) and 57 days (d57, post-treatment) after first treatment infusion by enzyme-linked immunosorbent assay (ELISA): Quantikine Immunoassays (R&D Systems) were used according to the manufacturer’s instructions.

Additionally, tissue samples (baseline and at surgery) were assessed for MVD by immunohistochemistry, as described previously [27].

### Statistical analysis

The primary end-point was pathological complete response (pCR), as defined by ypT_0_N_0_. Based on previous trials, a conservative estimate of pCR rate for patients with LARC treated with capecitabine and radiation is approximately 15% [28-35]. Following the SWE method for randomized phase II clinical trials [36], assuming a minimum pCR rate of at least 15% in one of the arms, a difference between the two arms of 10%, and accepting a probability of correct selection of 87%, 41 pts per arm were needed. Considering a 10% of non-evaluable patients, the study needed to enroll a minimum of 90 patients.

The primary efficacy analyses were conducted on an intention to treat (ITT) basis (all randomized patients). The safety analysis was performed for the safety population (patients who initiated trial therapy). The statistical analyses were performed using SAS version 9.2.

The pCR rate was calculated and the 95% confidence intervals (CI) were estimated using normal approximation. Secondary end-points were safety, and rates of downstaging (lower ypT compared with the pretreatment clinical T), sphinctersparing surgery, local recurrence, post-surgical complication and of complete resection (R0). Proportions were compared using a χ^2^ test or, if this could not be used, a Fisher’s exact test.

All statistical tests were two-sided. The significance level was established at a value of α =0.05.

The intra-individual differences at different time points for the concentrations of biomarkers were tested through paired t-Test if the population was normally distributed, or else through Wilcoxon signed rank test. To test these differences between groups of treatment, Student test was used if the distribution was normal, and Mann–Whitney test if it was not normally distributed.

Logistic regression model was adopted to estimate and test the biomarkers for their association with downstaging. Results were expressed as odds ratios and their 95% CI. Data analysis is reported according to REMARK guidelines [37].

## Results

**Figure 1:**
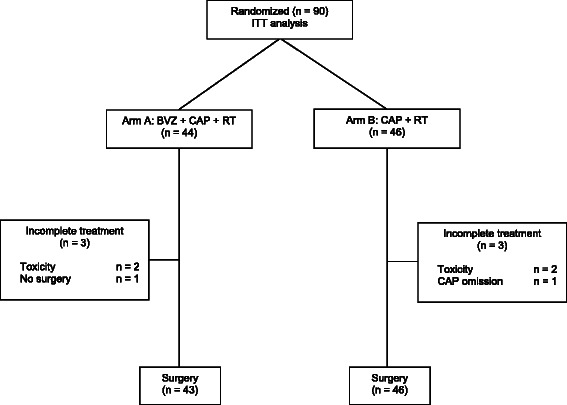
**CONSORT diagram.**

Between December 2009 and March 2011, 90 patients were randomly assigned through 12 Spanish hospitals, 44 in arm A and 46 in arm B (Figure 1).

Baseline patient characteristics were well balanced between groups (Table 1). The median distance from anal verge was 6.5 cm in arm A and 7.0 cm in arm B.

**Table 1:** **Baseline characteristics: intention to treat population (n = 90)**

	Arm A		Arm B	
	(BVZ + CAP + RT)		(CAP + RT)	
	(n° patients = 44)		(n° patients = 46)	
Parameter	No. of patients	%	No. of patients	%
Sex				
Male	25	57	30	65
Female	19	43	16	35
Age, years				
Median	64		60	
Range	37-77		42-78	
ECOG				
0	22	50	30	65
1	22	50	16	35
Tumor location				
Upper third	10	23	10	22
Middle third	14	32	19	41
Lower third	20	45	16	35
Unknown	0	0	1	2
Clinical tumor category				
T_2_	1	2	1	2
T_3_	33	75	38	83
T_4_	10	23	7	15
Clinical nodal category				
N_0_	7	16	5	11
N_1_	18	41	27	59
N_2_	19	43	14	30
Clinical TNM				
IIA	6	14	5	11
IIB	1	2	0	0
IIIB	18	41	27	59
IIIC	19	43	14	30

### Treatment compliance

Treatment compliance was similar in both arms. Forty-one (93%) and forty-three (93%) patients completed the planned CRT treatment in arm A and arm B, respectively.

Five patients received a dose of RT of lower than 45 Gy, 3 in arm A (1 of them discontinued the treatment after receiving 30.6 Gy, due to toxicity) and 2 in arm B. Two patients in arm A and three patients in arm B received <3 cycles of capecitabine, due to toxicity; a dose reduction of capecitabine was performed in 1 patient (toxicity). In arm A, all but one patient (toxicity) received the planned 3 cycles of bevacizumab.

#### Safety

Treatment-related toxicity occurring at a frequency >10% of patients is summarized in Table 2. The overall rate of patients with grade 3 to 4 treatment-related toxicity was 16% in arm A versus 13% in arm B (*p* =0.70). There was no grade 3 or greater hematological toxicity. Three patients in arm A (grade ≤ 2) and two in arm B (one grade 1 and another grade 3) had hypertension, two of them considered as probably related to the study treatment (arm A).

**Table 2:** **Early adverse events related** ^**a**^ **to treatment (≥10%) per patient (%) according to NCI-CTC criteria v3.0**

	Arm A		Arm B	
	(BVZ + CAP + RT)		(CAP + RT)	
	(n° patients = 44)		(n° patients = 46)	
	% grade 1/2	% grade 3	% grade 1/2	% grade 3
Astenia/fatigue	55	2	24	-
Diarrhea	36	-	41	-
Dysuria	18	-	30	-
Rectal tenesmus	18	-	17	-
Nausea	13	-	11	-
Hand-foot syndrome	14	-	4	2
Anorectal discomfort	9	2	9	-
Anorexia	9	-	11	-

Note: there were no grade 4 events.

^a^An adverse event was considered attributable to bevacizumab, capecitabine or radiation if it was deemed remotely, possibly or probably related.

Surgery was performed after a median interval of 51 days (range, 36–100 days). All included patients but one (arm A: peritoneal carcinomatosis) proceeded to surgery. Anterior resection and abdominoperineal resection were performed in 27 (61%) and 15 patients (34%) in arm A and in 31 (67%) and 13 patients (28%) in arm B, respectively: other procedures were performed in the remaining patients. Thirty-four (77%) patients in arm A and 36 (78%) in arm B underwent TME: the remaining patients undergoing surgery (9 in arm A and 10 in arm B) had a partial mesorectal excision (PME) due to their tumors were located at the upper rectum. Sphincter preservation was achieved in 27 (61%) and 31 (67%) patients in arm A and B, respectively. The overall rate of surgical complications was not significantly different between groups. Two patients in arm A and 6 patients in arm B experienced local complications. Nineteen patients (43%) and 18 (39%) patients in arm A and B experienced at least one postoperative complication, respectively. Ten patients (7 in arm A (15.9%) and 3 in arm B (6.5%)) required reoperation, due to anastomotic dehiscence. There were no perioperative deaths. During surgery, distant metastases in abdomen were found in 4 patients, all of them in arm B.

#### Response to treatment

The ypCR (ypT_0_N_0_) rate in the ITT population was 16% (7/44 patients; 95% CI 7-31%) in arm A and 11% (5/46 patients; 95% CI 4-24%) in arm B (*p* =0.54). Absence of residual tumor (R0) was achieved in all except 3 patients, who had microscopic residual disease (R1). Sixteen (36%) patients in arm A and 20 (44%) patients in arm B attained Mandard tumor regression grade (TRG) 1 or 2 responses. A decrease in the T stage (T downstaging) was achieved by 26 (59%) of 43 operated patients in arm A versus 18 (39%) of 46 in arm B (*p* =0.04) (Table 3).

**Table 3:** **T and N downstaging (intention to treat population: 90 patients)**

	Arm A		Arm B		*p*value
	(BVZ + CAP + RT)		(CAP + RT)		
	(n° patients = 44)		(n° patients = 46)		
Parameter	No. of patients	%	No. of patients	%	
ypT					***0.0429***
Better	26	59	18	39	
Equal	16	37	28	61	
Worse	1	2	0	0	
Missing^a^	1	2	0	0	
ypN					0.0865
Better	24	55	35	76	
Equal	15	34	8	18	
Worse	4	9	2	4	
No Evaluable^b^	0	0	1	2	
Missing^a^	1	2	0	0	
ypT and ypN					0.5612
Better in both	18	41	16	35	
Better in one of them	14	32	20	43	
Worse in both	11	25	9	20	
No Evaluable^b^	0	0	1	2	
Missing^a^	1	2	0	0	

^a^No surgery.

^b^It was reported as ypNx.

After a median follow-up of 18 months (range, 3–28 months), 88 (98%) patients remained alive, and 73 (81%) patients continued to be free of any sign of disease. One patient in arm B had a local recurrence, six patients in each arm developed distant metastases, four patients in arm B presented a second tumor (one of them was the patient with local recurrence), and two patients in arm A had died due to the underlying cancer (one of them had presented distant metastases).

#### Study of prognostic factors

**Figure 2:**
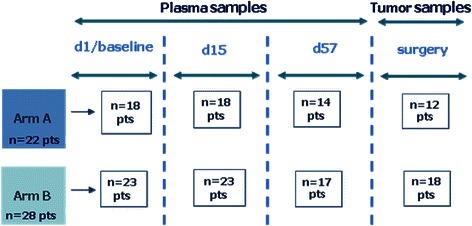
**Plasma and tumor samples availability for biomarker analyses.**

At the time of analysis, biomarker outcome data were available for 50 of the 90 randomized patients (56% of trial participants), 22/44 (50%) treated in arm A and 28/46 (61%) in arm B. At least paired plasma samples were available for 18 patients in arm A and 23 in arm B; tumor samples (pretreatment and from surgical specimen) were available from 12 and 18 patients in arm A and B, respectively (Figure 2).

Downstaging was evaluated in 49 out of the 50 patients included in the sub-study: one patient did not undergo surgery. Eleven patients in each arm were downstaged: no statistically significant differences were observed between groups (*p* =0.36).

No differences were observed in baseline levels of any biomarker between both arms. The pretreatment level of biomarkers showed no association with downstaging.

Angiopoietin-2 levels were significantly higher in arm B than in arm A at d15 (*p* =0.0056) and d57 (*p* =0.0133) (Table 4). Angiopoietin-2 levels significantly decreased in arm A at d15 (*p* =0.04) and increased in arm B (*p* =0.01) at d57. When intergroup differences were studied, plasma Ang-2 levels decreased in arm A and increased in arm B at all time points compared to baseline level, with significant differences between levels in group A and B at all time points. Overall, decrease in Ang-2 levels from baseline to d57, was significantly associated with tumor downstaging (OR: 0.95, 95% CI 0.91-0.99; *p* =0.02).

**Table 4:** **Evolution of biomarkers during the study compared to pretreatment (basal) value**

	Arm A				Arm B			
	(BVZ + CAP + RT)				(CAP + RT)			
	*VEGF*	*VEGFR-2*	*Ang-2*	*MVD* ^b^	*VEGF*	*VEGFR-2*	*Ang-2*	*MVD* ^b^
Basal								
n	18	18	18	12	23	23	23	18
Median	56	9056	2424	14	85	9282	3609	14
*p*	NA	NA	NA	NA	NA	NA	NA	NA
D15^a^								
n	18	18	18	12	23	23	23	18
Median	13	7	**−**22	**−**8	**−**40	4	16	34
*p*	0.19	0.18	***0.04***	0.73	0.11	0.33	0.11	***0.03***
D57^a^								
n	14	14	13	NA	17	17	17	NA
Median	55	7	**−**16	NA	**−**13	**−**2	20	NA
*p*	***0.03***	0.20	0.20	NA	0.71	0.86	***0.01***	NA

*Abbreviations*: *IQR* interquartile range, *VEGF* vascular endothelial growth factor, *VEGFR-2* circulating soluble VEGF receptor 2, *Ang-2* angiopoietin-2.

^a^Percent difference (%) = [(Later value- Basal value)/Basal value] × 100.

^b^Tissue samples (baseline and at surgery).

In contrast to serum Ang-2, VEGF levels increased in arm A and decreased in arm B at all time points compared to baseline levels, with significant intergroup differences at all time points. Nevertheless, there was no significant association between serum VEGF levels and downstaging.

There were no significant changes in other biomarker levels and none was associated with tumor downstaging.

## Discussion

This study confirms the feasibility of preoperative CRT with bevacizumab and capecitabine in patients with LARC, in a randomized trial. Although the patients who received bevacizumab tended to have a higher pCR rate, the predefined efficacy endpoint was not met. Interesting, Ang-2 plasma levels significantly decreased along the study in patients receiving bevacizumab and, overall, decrease in Ang-2 levels was significantly associated with tumor downstaging: those findings suggest improved tumor shrinkage related to the use of bevacizumab and a potential role of plasma Ang-2 to monitor downstaging.

We conducted this study to further understand the role of bevacizumab in the treatment of LACR. The 5 mg/kg dose was chosen based on the toxicity observed in the Willet et al. study [18], and the Xeberecto Trial (back to back submission [12]), a phase II study of preoperative bevacizumab, capecitabine and radiotherapy for resectable LARC. Our trial was initially designed to include oxaliplatin in both arms; however because the results of two large randomized studies [38,39] did not demonstrate the benefit of oxaliplatin with concurrent irradiation, the study was amended to do not use this drug.

Our patients had a relatively high risk for pelvic recurrence (98% were T3 or T4, and 87% were N+). The reached pCR rate of 16%, albeit not too high, is within the range (13-36%) reported across a number of phase II studies evaluating bevacizumab plus CRT (Table 5) [6-11]. However, caution is needed when comparing pCR rates as this item itself is highly dependent on the quality of the pathological examination [40].

**Table 5:** **Clinical trials of bevacizumab + radiochemotherapy as pre-operative treatment of locally advanced rectal cancer**

Author and regimen	No. of patients	pCR (yp T_0_-N_0_; %)	T-downstaging (%)	Grade 3 most common toxicities
Willet [11]				
BVZ + 5FU + RT	32	16^a^	50	Diarrhea and hypertension
Spigel [9]				
BVZ + 5FU + RT	35	29	NA	Diarrhea
Crane [6]				
BVZ + CAP + RT	25	32	64	Perianal desquamation
Velenik [10]				
BVZ + CAP + RT	61	13	45	Dermatitis
Kennecke [7]				
BVZ + CAP + OX + RT	42	18	NA	Diarrhea
Nogue [8]				
BVZ + XELOX → BVZ + CAP + RT	47	36	NA	Rectal tenesmus
Salazar (current study)				
BVZ + CAP + RT	44	16	26	Astenia/fatigue
CAP + RT	46	11	18	

*Abbreviations*: *pCR* pathological complete response, *BVZ* bevacizumab, *5FU* 5-fluorouracil, *CAP* capecitabine, *OX* oxaliplatin, *XELOX* capecitabine + oxaliplatin, *RT* radiotherapy, *NA* not available.

^a^ypT_0._

The decrease in the T stage was significantly higher in patients receiving bevacizumab, although the rate of downstaging in both arms was lower than in other single arm studies using preoperative bevacizumab plus CRT [6,10,11]. This should be interpreted with caution because of the preoperative MRI staging technical limitation and lack of central imaging evaluation.

Toxicities were expected and manageable. Early toxicity was mild in both arms. The most serious post-operative complication was anastomotic dehiscence, which occurred more frequently in patients treated with bevacizumab, but in the range reported by others [6,7,9-11].

Suitable biomarkers predicting patients who are likely to benefit from bevacizumab treatment remain elusive [41]. In our exploratory analyses decrease in Ang-2 levels from baseline to d57 was significantly associated with tumor downstaging. Moreover, Ang-2 levels decreased in the bevacizumab arm and increased in the other arm at all time points compared to baseline levels, with significant differences between levels in both groups at all time points. Angiopoietin-2 has been proposed as a gatekeeper of VEGF function and vascular remodeling [42,43], and has been shown to promote metastatic growth [44]. Goede et al. [14] found that serum levels of Ang-2 in patients with metastatic colorectal cancer were significantly higher than in healthy individuals: moreover, in that study, compared with high serum Ang-2 levels, low serum Ang-2 was associated with an outstanding response rate, better disease control and excellent overall survival (OS). Although one can only speculate about this relationship, it seems plausible that adding bevacizumab to standard CRT induces a decrease in serum Ang-2 levels that could facilitate tumor regression.

On the contrary, we found that serum VEGF levels increased in arm A and decreased in arm B at all time points compared to baseline levels, with significant intergroup differences at all time points: this finding had not influence on downstaging. Similarly, several studies have shown acute increases in circulating VEGF after the start of bevacizumab [11]. Nevertheless, changes in VEGF concentrations associated with bevacizumab treatment have not necessarily been predictive of benefit [41]. Detailed analyses are needed of total and free VEGF levels during treatment before circulating VEGF is dismissed as a biomarker.

In accordance with previous reports [11,12,14,45], pretreatment level of VEGF and tumor MVD was not correlated to clinical end points.

This trial has several limitations. Firstly, the selection of patients for the biologic sub-study was opportunistic, by including those patients who gave their consent and in which assessment of selected biomarkers was available at different points, although there is no reason to suspect any differences with other patients where those determinations were not possible. Secondly, the small size of each cohort in that study makes it difficult to find potential associations between changes in different biomarkers, and comparisons between both arms: nevertheless, our findings point in the same direction as that described by other authors. Thirdly, TME was not possible in around 20% of patients, due to the tumor localization: as stated previously [5], TME was mandatory for tumors in the lower two-thirds of the rectum, with PME being permitted for those in the upper third, provided a distal margin of at least 5 cm without coning was observed. Finally, as in other studies realized in patients with LARC, 4 patients were found having distant metastases at the time of surgery, a number not very different of those published by others [39]. As we didn’t make a reevaluation of the baseline studies, we cannot discharge a possible mistake in the inclusion of any patient.

## Conclusions

The results of this randomized study support the data described previously in single arm studies about the feasibility of the addition of bevacizumab to a standard neoadjuvant capecitabine-based CRT regimen, as well as its potential role in downstaging. It will be also important to continue observation of these patients to elucidate long-term outcome and morbidity of this strategy. Furthermore, although definitive judgment on the role of Ang-2 as a specific biomarker of outcome to bevacizumab in LARC will require further analysis of larger numbers of patients from phase III trials, the results arising from this study should encourage researchers to further investigate the value of Ang-2 amongst others as a potential biomarker to monitor the added value of bevacizumab in clinically relevant endpoints.

## Abbreviations

5-FU: 5-fluorouracil
Ang-2: Angiopoietin-2
BEV: Bevacizumab
CAP: Capecitabina
CRT: Chemoradiotherapy
ECOG: Eastern Cooperative Oncology Group
ELISA: Enzyme-linked immunosorbent assay
ITT: Intention to treat
LARC: Locally advanced rectal cancer
MRI: Magnetic resonance imaging
MVD: Microvessel density
NCI-CTC: National Cancer Institute Common Toxicity Criteria
OS: Overall survival
pCR: Pathological complete response
pts: Patients
R0: Radical resection
RT: Radiotherapy
TME: Total mesorectal excision
TRG: Tumor regression grade
TTD group: Spanish Cooperative Group for the Treatment of Digestive Tumors
VEGF: Vascular endothelial growth factor
ypCR: Pathologic complete response
